# Adherence to prophylaxis and bleeding outcome: A multicenter Nigerian study

**DOI:** 10.1371/journal.pone.0264600

**Published:** 2023-02-02

**Authors:** Theresa Ukamaka Nwagha, Helen Chioma Okoye, Saleh Yuguda, Christiana Enefiok Udo, Mutiat Kehinde Ogunfemi, Dalhat Haliru Gwarzo, Nnamdi Joel Osuji

**Affiliations:** 1 Department of Haematology and Immunology, Southeast Haemophilia Treatment Centre, College of Medicine, University of Nigeria Ituku-Ozalla Campus, Enugu, Nigeria; 2 Department of Haematology and Blood Transfusion, Gombe State University/Federal Teaching Hospital Gombe, Gombe, Nigeria; 3 Department of Haematology, National Hospital Abuja, Abuja, Nigeria; 4 Department of Haematology and Blood Transfusion, University of Ilorin Teaching Hospital, Ilorin Kwara, Nigeria; 5 Department of Haematology, Aminu Kano Teaching Hospital, Kano, Nigeria; 6 Dental and Maxillofacial Surgery, Maitama Abuja, Nigeria; Para Federal University, BRAZIL

## Abstract

In Nigeria, low-dose prophylaxis is the standard of care as it reduces bleeding, development of target joints, arthropathy, and improvement of quality of life. Non-adherence or poor adherence can prevent the achievement of these outcomes. The levels and determinants of (non-)adherence among persons with haaemophilia (PWH) in Sub-Saharan Africa have not been evidenced. We aimed to evaluate self-reported adherence among PWH, provide evidence of determinants/predictors of adherence, and establish the associations between nonadherence and presence of target joints and annualized bleed rate. A cross-sectional survey of 42 participants on low-dose prophylaxis recruited during outpatient appointments in 5 haemophilia treatment centers in Nigeria. We used the validated Haemophilia Regimen Treatment Adherence Scale- Prophylaxis (VERITAS -Pro), 24 questions on six subscales (time, dose, plan, remember, skip, and communicate) questionnaire. The options of VERITAS -Pro were represented in a 5 Likert scale and the possible subscale ranged from 4 points (most adherent) to 20 points (least adherent) and the possible total score ranged from 24 (most adherent) to 120 (least adherent) the cutoff for overall adherence put at > 61 to indicate nonadherence. Information on the presence of target joints, the number of target joints, and annualized bleeding rates were collected from medical files. The mean age of the participants was 9.79 (6.29) years, with 96.6% having hemophilia A and 79.3% having target joints. Overall adherence to the prophylaxis regimen was 81.0%. The mean total VERITAS-Pro for the adherent group and the non-adherent group was 37.35 ±9.08 and 63.0± 6.37, respectively. The mean subscale scores for the adherent group ranged from 0.67 (communication) to 8.68 (planning), while the mean subscale scores range from 1.0 communication to 13.88 (planning) for the nonadherent group. The mean difference of all except the dosing subscale was statistically significant with p<0.05. Only the skipping subscale showed a statistically significant positive correlation with ABR in the non-adherent group p = 0.02. The findings indicate that adherence was very good, and most were in communication with their treatment centers. The skipping subscale was significantly associated with ABR for the nonadherent group. Interventions aimed at improving adherence are the key to better treatment outcomes. A multicenter study was needed to assess the reason for poor adherence.

## Introduction

In Haemophilia, prophylaxis is a treatment type which involves regular routine intravenous infusions of factor concentrate aimed at preventing anticipated bleeding episodes [1]. Prophylaxis as a standard of care in hemophilia was conceived from the observation that hemophilia patients with clotting factor level >1 IU/dl rarely experience spontaneous bleeding and have more conserved joint function [1]. Although evidence is available that prevention of arthropathy should require the achievement of higher factor levels, well above 1 IU/dL [2,3], benefits of prophylactic replacement of clotting factors in preventing bleeding and improving joint status and quality of life have been shown even when factor levels are not maintained above such levels [3].

Prophylaxis in children has better outcome when it is started at an early age [4]. The key to a successful long-term outcome in patients with hemophilia is an efficient prophylaxis that prevents bleeding in joints for children and adults with hemophilia. Even though prophylaxis does not reverse already established joint damage, it decreases frequency of bleeds and may slow deterioration of joint health with improve quality of life. Efficient prophylaxis requires taking into account the available resources which includes the clotting factor concentrate, trough levels, the bleeding trigger (activity levels, chronic synovitis, already existing arthropathy), and most importantly the number of acceptable bleeds, especially joint bleeds [5].

The treatment objectives can vary depending on the available resources in the respective countries and treatment centres. In countries with significant resource constraints, lower doses of prophylaxis given more frequently may be an effective option. The idea is to reduce the number of spontaneous bleeds in a bid to forestall the development and progress of joint arthropathy. Prophylaxis has greatly improved joint health and its challenging joint outcome assessment [5,6].

There are three types of prophylaxis primary, secondary and tertiary. Primary prophylaxis is the institution of a regular continuous prophylaxis before any documented joint disease, established clinically and/or using imaging studies, and before the second clinically evident joint bleed and the age of 3 years. The use of primary prophylaxis has allowed many children with severe hemophilia now to live more normal lives with fewer episodes of acute bleeds and decreased orthopedic complications. Whereas secondary prophylaxis is the regular continuous prophylaxis started after 2 or more joint bleeds but before the onset of joint disease which is usually at 3 or more years of age, tertiary prophylaxis is the regular continuous prophylaxis initiated after the onset of documented joint disease. Tertiary prophylaxis typically applies to prophylaxis commenced in adulthood [7].

How to start and when to start prophylaxis with either standard half-life (SHL) or extended half-life (EHL) coagulation factor concentrates (CFCs) are not significantly different. In both cases, prophylaxis should be commenced early by starting with a high-dose/high-frequency approach or a low-frequency approach, followed by escalation of frequency [1,7]. With EHL CFCs, less frequent infusions may be sufficient for many individuals, up to once weekly for patients with haemophilia A and even at longer intervals for patients with haemophilia B. As EHL CFCs must still be given intravenously, they remain difficult to administer in very young children with poor peripheral venous access [8]. With advances in haemophilia treatment, prophylaxis with a non-replacement agent (emicizumab, the FVIII-mimetic bispecific humanized monoclonal antibody) subcutaneously administered, has been proven to reduce the rate of bleeding even better than the CFCs [9,10]. Other agents with different non-replacement mechanisms are on clinical trials. However costs of non-replacement agents limit the availability for prophylaxis in low-income countries [11,12]. The objectives of prophylaxis in different situations vary depending on the patient’s age and underlying conditions [13]. Prophylaxis provides protection from other types of hemorrhages in hemophilia, including preventing or substantially reducing the risk of intracranial hemorrhage [14].

In resource-constrained countries, low-dose prophylaxis tends to focus on the use of smaller doses. This is a way for patients in these countries to start receiving prophylaxis but at lower cost. Cost is minimized by minimizing the doses used while maintaining similar frequencies of infusion [15]. In Nigeria low dose prophylaxis is the standard of care for haemophilia as it reduces bleed, development of target joint, haemophilic arthropathy, at the same time improve quality of life [16].

Despite the benefits of prophylaxis, adherence has traditionally saddled with significant challenges stemming from the burden of administering CFCs both intravenously and frequently, there is difficulty in obtaining a venous access, to child/family resistance to the time-consuming nature of conventional prophylaxis [2].

Non-adherence or poor adherence can prevent the achievement of the goals of prophylaxis. Adherence among person with haemophilia on prophylaxis in Sub-Sahara Africa has not been evidenced.

## Materials and methods

This was a cross-sectional multicenter study carried out in five (5) Haemophilia treatment centers (HTCs) in Nigeria over a period of 4 months. The participating institutions include University of Nigeria Teaching Hospital Enugu, University of Ilorin Teaching Hospital Ilorin, Aminu Kano Teaching Hospital Kano, National Hospital Abuja and Federal medical center Gombe. In these centres, the available treatment options were low-dose prophylactic regimen and the on-demand regimen. While most of the patients on prophylaxis received EHL replacement, those on on-demand therapy mostly received SHL products. Prophylaxis treatment was commenced in 2015 in Nigeria. Adherence to prophylaxis was measured using a validated Haemophilia Regimen Treatment Adherence Scale- Prophylaxis (VERITAS -Pro) [17], 24 questions on six (four-item) subscales (time, dose, plan, remember, skip, and communicate) questionnaire which was administered to patients or their caregivers.

Ethical consideration—approval for the study was gotten from the Research Ethics committee of the University of Nigeria Teaching Hospital. Informed written consent was obtained from all adults and guardian (for the children).

### Participants

Only participants who had given consent and had been on prophylaxis for a minimum of 12 months were eligible. A total of 42 patients on low dose prophylaxis of clotting factor concentrates (CFC) were consecutively recruited for this study over a period of 4-months using a convenience sampling method during their outpatient visits. Inclusion criteria were all patients with haemophilia A or B on prophylaxis with CFC (self- infused or given by the caregiver) in the last one year. Patients on “on-demand” therapy regimen and those not able to infuse at home were excluded from the study. Permission to access infusion log data was sought from individuals for the purpose of this study and the annualized bleeding rate (ABR) calculated for each participant. For patients less than 11 years, the questionnaire was administered through the help of the care givers, whereas those 11 year and above were allowed to answer by self. Participants were assured that all study- related data will be kept confidential by the study coordinator and identification will only be through an assigned unique number.

### Adherence measurement

The Validated Haemophilia Regimen Treatment Adherence Scale- Prophylaxis- (VERITAS-Pro) is a 24 -item questionnaire divided into six (6) sub scales: Time, Dose, Plan, Remember, Skip and Communicate. Response options are presented as five- point Likert scales ranging from ‘Always’ to ‘Never’; Always reflects best possible adherence for some items and the worst possible adherence for other items. Total scores range from 24–120, and subscale scores range from 4–20 with lower scores indicating higher adherence. The patient or caregiver was given the VERITAS-Pro and allowed as much time as necessary to complete the survey. Participants not returning the survey within a week received reminders via text messages or phone calls and were considered lost to follow-up after two unanswered phone calls/messages.

### Data analysis

Data collected were analyzed using the Statistical Package for Social Science (SPSS) software. Mean and standard deviations (descriptive statistics) were calculated for each subscale. Frequency and proportion were used to describe the categorical variables. Pearson correlation was used to analyze the association between self-reported adherence and annualized bleeding rate.

## Results

A total of 42 persons with haemophilia (PWH) on prophylaxis regimen from 5 Haemophilia treatment centers participated in the study. The majority (96.6%) of the patients had haemophilia A. Thirty-seven (88%) of them had moderate haemophilia while only 5 (12%) had severe disease. The age of the participants varied from 1 to 30 years (mean: 9.8 ± 6.3years). Thirty-three patients (79.3%) had target joints.

### Self-reported adherence

Adherence was defined as the total sum of all subscales <61 and nonadherence as the total sum of all subscales >61 based on the cutoff scores proposed by the original validation study^13^. There was a record of 81.0% adherence among the participants. For the subscales, 83.3% of the participants were more adherent in the timing of their prophylaxis and communication with the HTC, while the highest non-adherence,52.4% was recorded in the planning subscale (see Table 1).

**Table 1:** Adherence status in subscales.

Variable	Frequency	
Adherence Status	(n = 42)	Percent
Adherent	34	81.0
Non-adherent	8	19.0
Subscales	Adherent	Non-adherent
Timing	35 (83.3)	7 (16.7)
Dosing	31 (73.8)	11 (26.2)
Planning	20 (47.6)	22 (52.4)
Remember	39 (92.9)	3 (7.1)
Skipping	34 (81.0)	8 (19.0)
Communication	35 (83.3)	7 (16.7)

The mean logarithmic age was 9.77±1.62 and 4.90± 2.34 years, for the adherent group and non-adherent group, respectively; the difference was statistically significant with a p-value of 0.02. The difference between the mean ABR and the mean number of target joints between the adherent and non-adherent groups was not statistically significant with p values of 0.90 and 0.31, respectively. (See Table 2). Sub analysis of age groups (<18 years vs ≥18 years) did not show any significant difference in the overall adherence and all subscales (Table 3).

**Table 2:** Age, ABR and number of target joints in the adherent vs. non-adherent group.

Variable	Adhere				Non-Adherence					
	Mean	SD	Min	Max	Mean	SD	Min	Max	t (p-value)	M. Diff
**Age (Log)**	9.77	1.62	3.98	30.19	4.90	2.34	1.99	16.98	2.57 (0.016)	1.95
**Annualized Bleeding Rate (Log)**	8.12	2.95	1.99	60.25	7.59	6.17	1.99	60.26	0.13 (0.894)	1.09
**Number of Target joint (Log)**	2.57	1.35	1.99	3.98	2.00	1.00	1.99	1.99	1.08 (0.308)	1.29

**Table 3:** Adherence by age.

Variable		Age (months)		Bivariate Test
**Subscales**	**Adherent**	≤ 18	>18	(p-value)
**Timing**	Yes	15 (75.0)	8 (88.9)	0.633
	No	5 (25.0)	1 (11.1)	
**Dosing**	Yes	17 (85.0)	5 (55.6)	0.158
	No	3 (15.0)	4 (44.4)	
**Planning**	Yes	8 (40.0)	6 (66.7)	0.245
	No	12 (60.0)	3 (33.3)	
**Remember**	Yes	19 (95.0)	9 (100.0)	1.000
	No	1 (5.0)	0	
**Skipping**	Yes	17 (85.0)	8 (88.9)	1.000
	No	3 (15.0)	1 (11.1)	
**Communication**	Yes	17 (85.0)	8 (88.9)	1.000
	No	3 (15.0)	1 (11.1)	
**Overall**	Yes	15 (75.0)	8 (88.9)	0.633
	No	5 (25.0)	1 (11.1)	

### Overall Veritas Pro scores

The mean total VERITAS-Pro score for the adherence group for the total sample was 37.35 ± 9.08 with a range of 24 to 56 (Table 4). Subscale mean scores ranged from 0.67 (communication) to 8.6 (planning), indicating that participants reported the highest adherence to communicating with HTC and the lowest adherence to planning for administering prophylaxis. For the non-adherent group, Subscale mean scores ranged from 0.60 (communicating) to 10 (skipping), indicating that participants reported the highest adherence to communicating with HTC and the lowest adherence in skipping prophylaxis.

**Table 4:** Overall self-reported adhesion.

Variable	Adherence							Non-Adherence								
	N	Mean	SD	Min	Max	Skew	Kurt	N	Mean	SD	Min	Max	Skew	Kurt	t (p-value)	M. Diff
**Self-Reported Adherence**																
Timing	34	0.78	0.17	0.60	1.23	0.67	-0.36	8	1.02	0.13	0.85	1.15	-0.16	-2.33	-4.43 (0.001*)	-0.24
Dosing	34	5.12	1.68	4	9	1.30	0.22	8	6.25	2.49	4	10	0.29	-1.98	-1.56 (0.127)	-1.13
Planning	34	8.68	3.23	4	17	0.68	0.43	8	13.88	4.49	5	20	-1.08	1.76	-3.10 (0.013*)	-5.19
Remembering	34	5.62	1.94	4	10	0.95	-0.48	8	8.25	244	6	13	1.04	0.91	-2.85 (0.019*)	-2.63
Skipping	34	6.50	2.53	4	13	0.73	-0.38	8	12.88	2.64	10	17	0.49	-1.13	-6.19 (<0.001*)	-6.38
Communicating	34	0.67	0.13	0.60	1.15	2.16	4.82	8	1.00	0.18	0.60	1.15	-1.85	3.54	-4.91 (0.001*)	-0.34
Total	34	37.35	9.08	24	56	0.46	-0.61	8	63.00	6.37	58	78	2.30	5.88	14.59 (<0.001*)	-25.65

SD = standard deviation

* statistical significance.

There was a significant difference in the overall VERITAS-Pro score between the adherence group and the non-adherent group, p<0.01. For the subscale, there was a significant difference in timing, planning communication, skipping, and remembering with p< 0.05 See Table 4.

### Correlation analysis of self-reported adherence, annualized bleeding rate, and number of target joint

The association between self-reported adherence and annualized bleeding rate was tested with Pearson’s correlation, which revealed that there were non-statistically significant associations with subscales between the group except for ‘Skipping’ among the non-adherent group, with a significantly high positive correlation coefficient of 0.94 and a p-value of 0.018; See Table 5.

**Table 5:** Association between self-reported adherence, annualized bleeding rate, and number of target joint.

Variable		Timing	Dosing	Planning	Remembering	Skipping	Communicating	Sum
**Annualized Bleeding Rate (Log)**	**Adherent**	-0.19 (0.415)	-0.27 (0.231)	-0.25 (0.270)	-0.41 (0.067)	-0.04 (0.852)	-0.19 (0.416)	-0.35 (0.118)
	**Non-Adherent**	-0.28 (0.651)	0.64 (0.245)	-0.28 (0.643)	0.19 (0.760)	0.94 (0.018)*	-0.61 (0.270)	-0.41 (0.492)
	**Both**	-0.19 (0.358)	-0.03 (0.895)	-0.25 (0.224)	-0.28 (0.165)	0.16 (0.434)	-0.26 (0.209)	-0.22 (0.278)
**Number of Target Joint (Log)**	**Adherent**	-0.19 (0.617)	-0.50 (0.171)	-0.35 (0.361)	-0.46 (0.216)	-0.08 (0.846)	0.02 (0.958)	-0.35 (0.358)
	**Non-Adherent**	-	-	-	-	-	-	-
	**Both**	-0.31 (0.356)	-0.47 (0.147)	-0.32 (0.335)	-0.52 (0.101)	-0.33 (0.328)	-0.15 (0.658)	-0.47 (0.144)

## Discussion

Prophylaxis is the standard of care in the management of haemophlia and has been linked to superior outcomes achieved in patients with adequate adherence to treatment [18–20]. Adherence among PWH in Nigeria and in sub-Saharan Africa has not been evidenced so we designed this study to evaluate self-reported adherence among PWH and their determinants. Our study shows a high adherence status of 81% among PWH on prophylaxis. While it corroborates the findings from other researchers who reported similar adherence rates, it is contrary to some others that recorded either higher or lower adherence rates [21–23]. The disparities may be due to differences in health care models. Whereas the Spanish health system is that of universal free access to health care which may make them less compliant to treatment protocol [24], Nigerian patients pay out-of-pocket for most health care services and are more likely to feel the financial burden directly. The treatment protocol used may also be a factor. Dover et al provided evidence to show that PWH with less frequent infusions/week were more likely to adhere to treatment protocol than those who receive more frequent dosing per week [25]. We use a two-dose-per-week dosing for our PWH on low dose prophylaxis Knowledge also influences the level of adherence to medications [26]. Haemophilia treatment centres whose health care professionals are more knowledgeable about the dosing, dosage and effects of the medications are more likely to have high adherence than those who are not because they are more likely to impart positively to medication compliance by recommending a better protocol and handling drug-related problems [26]. Even though we did not assess knowledge, it could have accounted for the differences in level of adherence among different population.

The total mean score for the VERITAS-Pro subscales is 37.35 and 63.0 for the adherence and non-adherence groups respectively. This is comparable to previous studies. Similarly, the subscales “timing”, “skipping”, “planning”, “remembering”, and “communication” where significantly different between groups. Only “dosing” did not differ suggesting that those in non-adherence group could have skipped infusion, failed to maintain stock at home, had poor communication with their treatment centres, or altered their dosing timing, but maintained the recommended dose of infusion. a non-factor replacement agent, i.e emicizumab, is currently being used for prophylaxis in some settings [9,10]. It may improve adherence as it offers the convenience of once a week, 2 weekly or 4 weekly dosing by subcutaneous administration. Other agents with different non-replacement mechanisms and prophylaxis regimens (dosing/frequency of administration) are on clinical trials [11,12]. These approaches are not associated with a peak and trough effects and are not measurable with standard laboratory methods, however they provide a protection comparable to factor levels of patients with mild hemophilia [11,12]. Overall, non-replacement agents could be very useful to facilitate early implementation and long-term management of prophylaxis, thus improving adherence particularly in the case of problems of venous access, like in children. However, economic constraints may make it not available in all countries, particularly in low-income ones.

Planning, one of the VERITAS-Pro subscales had the least frequency among the PWH in adherence group, only 20 (47.6%) of the adherence group had good planning, the problem could stem from not having enough factor at home and not keeping track of what is left to eventually running out of factors. More efforts should be made to maintain optimal stock of factors at home. For the non-adherent group, the subscale “remember” was the least. This indicates non-intentional non-adherence and it is similar to the work done by van Os et al. [21] The problem could have been forgetting to infuse the available factor even though they generally had poor planning among other subscales.

Interestingly, we observed that the mean age for those in adherence group was significantly higher than those in non-adherence group which may suggest that within our cohort of PWH, adherence is poorer among the younger age group. However, when we categorized the PWH into 2 groups; the children (<18 years) and the adults (≥ 18 years), there was no observed difference in adherence. This is contrary to the reports by previous studies conducted among PWH [27]. Prophylaxis is usually delivered to the paediatric/younger age group by caregivers. In our own case, the disparity may be a function of caregiver burden and economic strain which were not assessed [8]. Challenges of the caregiver in balancing the care offered to the child versus other family members as well as social needs could have played out in our study. Other possible reason supporting a lower adherence in younger children in our study could be that of venous access [28]. Securing a venous access in children is more challenging than that of adults or older children and obtaining a central line access is not a common practice among our patients as both caregivers and patients are not favourably disposed to it.

The purpose of instituting prophylaxis in haemophilia management is to prevent symptoms and attendant complications. There is evidence to support that PWH have reduced bleeds, pains, absenteeism, and improved quality of life. Our study however did not show any difference in the mean ABR and number of target joints between the adherence and non-adherence groups. Even though most patients on prophylaxis received EHL products as practiced in our cohort, we did not get the details of the exact product each of these participants received. This could have also impacted on the relationship between adherence to prophylaxis and ABR. In a similar study on paediatric patients, Krishnan reported no difference in ABR between the adherence and non-adherence group. All the participants had at least one bleeding episode and a target joint in the preceding year regardless of being on prophylaxis. Despite the lack of association between adherence and ABR, the skipping subscale showed a relationship such that targeting the skipping component may lead to improvements in ABR.

Skipping may suggests that the PWH were intentional about non-adherence [29]. For instance, PWH may skip infusion in the absence of bleeds and still score for skipping in the VERITA-Pro study. Only subscale “skipping” showed an association with adherence for ABR. The subscales “timing”, “dosing”, “planning”, “remembering”, and “communication” did not show any association with adherence status for both ABR and number of target joints. This may be due to variations in daily routine [25]. PWH may make adjustments in their dosing without communicating with the treatment centre, for example, a dose slated to be taken in the morning may be self-rescheduled for evening to accommodate personal program.

The results of this cross-sectional study should be interpreted in the light of its limitations. Being a self-reported adherence study, participants may be faced with recall bias and must have missed to accurately report some clinical outcomes. Our inability to separate the PWH into paediatrics, young adolescents, and adults due to small sample size may pose a limitation. A further stratification of our study participants into groups, for instance, <6 year, 6–12 years, 12–18 years etc. would have given us a better insight to the adherence. This was not possible in our study because of the small sample size which may leave one or two patients alone in one group. Additionally, we did not report on the severity of haemophilia, specific type of replacement factor they received, duration of prophylaxis and their correlation with adherence. We only reported on ABR since it is regarded as an important research endpoint which serves as a proof of efficacy, this notwithstanding, reporting ABR may be saddled with a significant variation and bias [30].

### Conclusion

The findings indicate that adherence was good, and most were in communication with their treatment centers. The skipping subscale was significantly associated with ABR for the nonadherent group. Interventions aimed at improving adherence are the key to better treatment outcomes. A robust multicenter study is needed to further investigate clinical correlations of adherence and assess the reason for poor adherence.

## Supporting information

S1 File (XLSX)

## References

[1] Sidharian N, Sude van R, Narayana Pillai V, Mattew S, Raj M, Viswam D, et al. Low dose prophylaxis for children with haemophilia in a resource limited setting in south India–a clinical audit report. Haemophilia. 2017; 23:e382–84. doi: 10.1111/hae.13272 28544055

[2] Löfqvist T, Nilsson IM, Berntorp E, Pettersson H. Haemophilia prophylaxis in young patients: a long-term follow-up. J Intern Med 1997;241:395–400. doi: 10.1046/j.1365-2796.1997.130135000.x 9183307

[3] Oldenburg J. Optimal treatment strategies for haemophilia: achievements and limitations of current prophylactic regimens. Blood. 2015; 125: 2038–44. 25712992 10.1182/blood-2015-01-528414

[4] Astermark J, Petrini P, Tengborn L, Schulman S, Ljung R, Berntorp E. Primary prophylaxis in severe haemophilia should be started at an early age but can be individualized., Br J Haematol, 1999, vol. 105 4(pg. 1109–1113). doi: 10.1046/j.1365-2141.1999.01463.x 10554828

[5] Manco-Johnson MJ, Abshire TC, Shapiro AD, et al. Prophylaxis versus episodic treatment to prevent joint disease in boys with severe hemophilia., N Engl J Med, 2007, vol. 357 6(pg. 535–544). doi: 10.1056/NEJMoa067659 17687129

[6] Fischer K, Steen Carlsson K, Petrini P, et al. Intermediate-dose versus high-dose prophylaxis for severe hemophilia: comparing outcome and costs since the 1970s., Blood, 2013, vol. 122 7(pg. 1129–1136). doi: 10.1182/blood-2012-12-470898 23777770 PMC3744988

[7] Blanchette VS, Key NS, Ljung LR, et al. Definitions in hemophilia: communication from the SSC of the ISTH. J Thromb Haemost. 2014;12(11):1935–1939. doi: 10.1111/jth.12672 25059285

[8] Fischer K, Collins PW, Ozelo MC, Srivastava A, Young G, Blanchette VS. When and how to start prophylaxis in boys with severe hemophilia without inhibitors: communication from the SSC of the ISTH. J Thromb Haemost. 2016;14(5):1105–1109. doi: 10.1111/jth.13298 27186714

[9] Mahlangu J, Oldenburg J, Paz-Priel I, Negrier C, Niggli M, Mancuso ME, et al. Emicizumab prophylaxis in patients who have haemophilia A without inhibitors. N Engl J Med. 2018; 379: 811–822. 30157389 10.1056/NEJMoa1803550

[10] Oldenburg J, Mahlangu JN, Kim B, Schitt C, Callaghan MU, Young G, et al. Emicizumab prophylaxis in haemophilia A with inhibitors. N Engl J Med. 2017; 377: 809–818. 28691557 10.1056/NEJMoa1703068

[11] Batorova A, Boban A, Brinza M, Lissitchkov T, Nemes L, Preloznik IZ, et al. Expert opinion on current and future prophylaxis therapies aimed at improving protection for people with haemophilia. J Med Life. 2022; 15: 570–78. 35646171 10.25122/jml-2022-0103 PMC9126455

[12] Fassel H, McGuinn C. Haemophilia: factoring in new therapies. *Br J Haematol*. 2021;194:835–50. doi: 10.1111/bjh.17580 34322873

[13] Nwagha TU, Okoye HC, Udo CE, Yuguda S, Korubo KI, Adeyemo TA. Clinical audit of low dose prophylaxis program for Nigerian children with haemophilia. West Afr J Med. 2022; 39: 11–15. 35156361

[14] Blanchette VS, Key NS, Ljung LR, Manco-Johnson MJ, van den Berg HM, Srivastava A. Subcommittee on Factor VIII, Factor IX and Rare Coagulation Disorders.

[15] Andersson NG, Auerswald G, Barnes C, et al. Intracranial haemorrhage in children and adolescents with severe haemophilia A or B—the impact of prophylactic treatment. Br J Haematol. 2017;179(2):298–307. doi: 10.1111/bjh.14844 28699675

[16] Gouider E, Jouini L, Achour M, et al. Low dose prophylaxis in Tunisian children with haemophilia. Haemophilia. 2017;23(1):77–81. doi: 10.1111/hae.13048 27943510

[17] Duncan N, Kronenberger W, Roberson C, Shapiro A. VERITAS-Pro: a new measure of adherence to prophylactic regimens in Haemophilia. 2010;16(2):247–55. doi: 10.1111/j.1365-2516.2009.02129.x . 19925631

[18] Khawaji M, Astermark J, Berntorp E. Lifelong prophylaxis in a large cohort of adult patients with severe haemophilia: a beneficial effect on orthopaedic outcome and quality of life. Eur J Haematol 2012; 88: 329–35. 8. doi: 10.1111/j.1600-0609.2012.01750.x 22221195

[19] Mondorf W, Kalnins W, Klamroth R. Patient-reported outcomes of 182 adults with severe haemophilia in Germany comparing prophylactic vs. on-demand replacement t therapy. Haemophilia 2013; 19: 558–63. 9. 23560639 10.1111/hae.12136

[20] Krishnan S, Vietri J, Furlan R, Duncan N. Adherence to prophylaxis is associated with better outcomes in moderate and severe haemophilia: results of a patient survey. Haemophilia (2015), 21, 64–70. doi: 10.1111/hae.12533 25470071

[21] De Moerloose P, Urbancik W, Van Den Berg HM, Richards M. A survey of adherence to haemophilia therapy in six European countries: results and recommendations. Haemophilia: the official journal of the World Federation of Hemophilia. 2008;14(5):931–8. doi: 10.1111/j.1365-2516.2008.01843.x 18684125

[22] Lindvall K, Colstrup L, Wollter IM, Klemenz G, Loogna K, Gronhaug S, et al. Compliance with treatment and understanding of own disease in patients with severe and moderate haemophilia. Haemophilia: the official journal of the World Federation of Hemophilia. 2006;12(1):47–51. doi: 10.1111/j.1365-2516.2006.01192.x 16409174

[23] Hacker M, Geraghty S, Manco-Johnson M. Barriers to compliance with prophylaxis therapy in haemophilia. Haemophilia. 2001;7(4):392–6. doi: 10.1046/j.1365-2516.2001.00534.x 11442644

[24] Cuesta-Barriuso R, Torres-Ortuño A, Galindo-Piñana G, Nieto-Munuera J, Duncan, López-Pina JA. Patient Preference and Adherence 2017:11 653–660. 28392680 10.2147/PPA.S126828 PMC5375636

[25] Dover S, Blanchette VS, Wrathall D, Pullenayegum E, Kazandjian D, Song B, et al. Haemophilia prophylaxis adherence and bleeding using a tailored frequency-escalated approach: the Candadian haemophilia primary prophylaxis study. Res Pract Thromb Haemost. 2020; 4: 318–25. 32110763 10.1002/rth2.12301 PMC7040543

[26] Mokhtar FM, Sathar J, Huri HZ. Medication adherence for haemophilia patients: outcome of prophylaxis treatment intervention. Healthcare (Basel) 2021; 9: 1702. doi: 10.3390/healthcare9121702 34946428 PMC8701723

[27] Schrijvers LH, Schuurmans MJ, Fiscer K. Promoting self-management and adherence during prophylaxis: evidence-based recommendations for haemophilia professionals. Haemophilia. 2016; 1–8. doi: 10.1111/hae.12904 27075653

[28] Thomburg CD, Duncan NA. Treatment adherence in haemophilia. Patient Prefer Adherence. 2017; 11: 1677–86. 29033555 10.2147/PPA.S139851 PMC5630068

[29] van Os SB, Troop NA, Sullivan KR, Hart DP (2017) Adherence to Prophylaxis in Adolescents and Young Adults with Severe Haemophilia: A Quantitative Study with Patients. PLoS ONE 12(1): e0169880. doi: 10.1371/journal.pone.0169880 28103266 PMC5245860

[30] Keipert C, Muller-Olling M, Gauly F, Arras-Reiter, Hilger A. Annual bleeding rates: pitfalls of clinical trial outcomes in haemophilia patients. Clin Transl Sci. 2020; 13: 1127–36. 32472976 10.1111/cts.12794 PMC7719362

